# Therapeutic Potential of Ginsenosides in Anthracycline-Induced Cardiotoxicity

**DOI:** 10.3390/molecules30122527

**Published:** 2025-06-10

**Authors:** Rongrong Bai, Zhigao Zhao, Xing Han, Mingying Shang, Guangxue Liu, Feng Xu, Shaoqing Cai

**Affiliations:** 1 Medical College, Xizang University, Lasa 850002, China; bairr012345@163.com; 2 College of Life Science and Engineering, Southwest Jiaotong University, Chengdu 610031, China; zhigaozhao@my.swjtu.edu.cn; 3 State Key Laboratory of Natural and Biomimetic Drugs, School of Pharmaceutical Sciences, Peking University, Beijing 100191, China; 2011110096@stu.pku.edu.cn (X.H.); myshang@bjmu.edu.cn (M.S.); guangxl@bjmu.edu.cn (G.L.)

**Keywords:** anthracyclines, anthracycline cardiotoxicity, cardiotoxicity, ginsenosides, oxidative stress, cardiovascular diseases

## Abstract

Anthracyclines play an irreplaceable role in cancer treatment, although their clinical application is limited due to severe side effects such as arrhythmia, cardiomyopathy, and myocardial infarction. The currently available clinical drugs for treating anthracycline-induced cardiotoxicity (AIC) are limited by numerous drawbacks, including the side effects of the therapeutic agents, single treatment mechanisms, and individual patient variations. Therefore, novel drugs with broader applicability and multitarget synergistic protective effects are, therefore, urgently needed. Ginsenosides, the primary bioactive constituents of plants belonging to the genus *Panax* (family *Araliaceae*), exhibit a wide range of pharmacological activities, including anti-inflammatory, antioxidative, and antitumor effects, and have demonstrated cardioprotective properties against AIC. This article examines the mechanisms of AIC and the modulatory effects of ginsenosides on these mechanisms. This review highlights the potential molecular targets and signaling pathways through which ginsenosides exert therapeutic effects on AIC, including the regulation of oxidative-stress-related pathways such as Keap1/Nrf2, MAPK, STAT, PI3K/Akt, and AMPK; the restoration of mitochondrial function; the modulation of autophagy; and the inhibition of pyroptosis, ferroptosis, and apoptosis. Therefore, this review serves as a theoretical basis and provides a research direction for future investigation regarding the prevention and treatment of AIC with ginsenosides, as well as clinical translation studies.

## 1. Introduction

Anthracyclines (ANTs) were discovered and applied in clinical treatment in the 1950s and 1960s and have since become a cornerstone in the treatment of various cancers, such as acute leukemia, lymphoma, and solid tumors, including breast cancer and gastric cancer [1]. Despite the continuous development of novel anticancer drugs in recent years, such as various targeted drugs and antibody–drug conjugates, ANTs are still used as drugs recommended in clinical guidelines for certain diseases. For example, the DA regimen (daunorubicin and cytarabine) is employed for the treatment of acute myeloid leukemia. The classic AC regimen (doxorubicin and cyclophosphamide) is used to treat triple-negative breast cancer [2,3]. ANTs exert potent anticancer effects through multiple mechanisms, such as embedding into the double strands of DNA, inhibiting DNA topoisomerase (DNA TOP) activity, and inducing free radical generation [4]. However, ANTs are broad-spectrum chemotherapeutic agents with poor specificity and exhibit several side effects alongside their antitumor efficacy, warranting consistent attention and urgent resolution of these concerns [5,6]. Anthracycline-induced cardiotoxicity (AIC) is one of the most severe adverse reactions and is characterized by diverse types, poor prognosis, and profound damage. Progressive emergence of AIC has been observed across various time points during treatment and recovery, with dose-dependent characteristics. Currently, the combination of ANTs and cardioprotective agents (dexrazoxane (DRZ) and statins) prevents the emergence of AIC according to the CSCO, EHA, and ESC guidelines [7,8]. Angiotensin-converting enzyme inhibitors (ACEIs), angiotensin receptor blockers (ARBs), and β-blockers are used as the primary treatment agents for AIC. In addition, improvements in the formulation of ANTs, such as liposomal encapsulation and nanopackaging, reportedly alleviate AIC [9]. However, although these therapeutic agents can reduce the incidence of AIC, drawbacks such as a short half-life, certain toxicity, and a potential impact on the efficacy of chemotherapy exist [8]. In summary, the single therapeutic mechanism of most drugs makes it difficult to comprehensively block the complex toxic pathways associated with ANTs. Therefore, novel AIC therapeutic agents with multitarget mechanisms, low toxicity profiles, and high universality are urgently needed.

**Figure 1:**
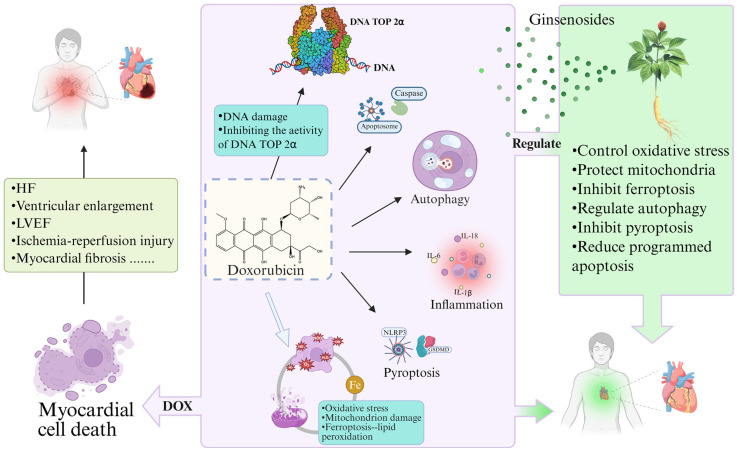
The toxic molecular mechanism of AIC and the regulatory role of ginsenosides.

In recent years, various natural plant components have been studied for use in the treatment of multiple diseases because of their broad therapeutic effects and low toxicity [10]. *Ginseng* is one such plant component that has demonstrated promising effects in terms of cardiac protection. Some clinical studies have shown that *ginseng* can relieve angina pectoris and improve cardiac function [11]. Ginsenosides, as the main active ingredients of *ginseng*, have pharmacological effects such as modulation of body immunity, antioxidative stress, and anti-inflammation, which provide new possibilities for the synergistic alleviation of AIC via multiple pathways [12,13,14]. A review of the relevant literature indicates that studies on the use of ginsenosides for the treatment of cardiovascular diseases (CVDs) have focused predominantly on conditions such as HF, ischemia–reperfusion injury (I/RI), atherosclerosis, myocardial infarction (MI), and arrhythmia [13,15,16]. In contrast, research specifically addressing the therapeutic potential of ginsenosides in AIC remains limited. Existing reviews on the cardioprotective mechanisms of ginsenosides—for both common CVDs and AIC—primarily emphasize their roles in mitigating oxidative stress, apoptosis, and dysregulated energy metabolism. Furthermore, several ginsenosides with potential therapeutic efficacy against AIC have been newly reported, yet their mechanisms of action—particularly those involving pyroptosis and ferroptosis—have not been comprehensively reviewed [17]. Therefore, this study begins by examining the specific molecular toxicological mechanisms underlying AIC. Second, it systematically summarizes how ginsenosides exert therapeutic effects through mechanisms such as oxidative stress modulation, mitochondrial function restoration, ion homeostasis regulation, inhibition of cell death pathways (including ferroptosis, pyroptosis, and apoptosis), and autophagy modulation (Figure 1). This review aims to provide a foundation for identifying novel therapeutic targets and agents for AIC while also advancing the pharmacological understanding of ginsenosides.

## 2. Mechanisms and Prevention of Anthracycline Cardiotoxicity

### 2.1. Classification of the AIC

AIC can be classified into different types, namely, acute, subacute, chronic, and late-onset cardiotoxicity, based on the duration of drug administration [8]. Acute and subacute cardiotoxicity generally emerge during the treatment or within two weeks post-treatment. Electrocardiogram monitoring reveals manifestations such as decreased voltage of the QRS complex, transient tachycardia, and prolonged QT interval [18], which are considered reversible lesions. Subacute toxicities, such as pericarditis–myocarditis syndrome, are relatively rare, although they are generally fatal, which is concerning [19]. Chronic cardiotoxicity emerges weeks or months after the completion of one year of chemotherapy and is characterized by cardiac enlargement, reduced LVEF, fatigue, dizziness, and other symptoms [20]. This side effect can prompt the progression of progressive HF to congestive HF. Delayed cardiac toxicity may emerge several years to even decades post-treatment and is characterized by late-onset cardiac dysfunction, conduction disturbances, and other such issues [21].

### 2.2. Mechanisms Associated with the AIC

#### 2.2.1. Oxidative-Stress-Mediated AIC

Oxidative stress is a physiological state in which cellular damage is caused by an imbalance between the production of reactive oxygen species (ROS; •O_2_^−^, •OH) and reactive nitrogen species (RNS; NO, •ONOO^−^) and the antioxidant capacity [22,23]. Excess ROS generation is induced by ANTs’ attack on proteins, nucleic acids, and other substances in cardiomyocytes, resulting in the induction of enzyme–protein inactivation and DNA breaks [24]. ROS can also lead to lipid peroxidation when combined with lipid macromolecules to generate toxic substances such as malondialdehyde (MDA) [25]. Intracellular antioxidants, such as superoxide dismutase (SOD), catalase (CAT), glutathione peroxidase (GPx), and glutathione (GSH), can be activated by various signaling molecules and pathways to counteract this abnormal oxidative damage [26,27].

Doxorubicin (DOX) is a representative anthracycline drug that generates ROS through several pathways. DOX undergoes redox reactions under the action of enzymes such as NADPH oxidase (NOX), endothelial nitric oxide synthase (eNOS), and xanthine oxidase. The reaction-generated doxorubicinol and semiquinone radicals serve as metabolites that spontaneously transfer electrons to free •O_2_^−^, leading to the production of large amounts of •O_2_^−^ and H_2_O_2_ [28]. •O_2_^−^ and NO can produce excess •ONOO^−^. H_2_O_2_ produces strongly toxic hydroxyl radicals •OH in the presence of transition metals [29,30]. These free radicals may undergo lipid peroxidation to produce toxic substances such as MDA, which damage cardiomyocytes. DOX can attack the abundant mitochondria in cardiomyocytes and chimerize mitochondrial DNA (mtDNA) to interrupt the electron transfer process in the respiratory chain and generate ROS [31,32]. Multiple studies have demonstrated that the regulation of autophagy by ANTs during cardiotoxicity is dualistic [33,34]. On the one hand, mitochondrial damage caused by DOX activates the PINK1/Parkin pathway, promotes the conversion of LC3-I to LC3-II to induce the onset of mitochondrial autophagy, and removes damaged mitochondria to reduce oxidative stress and promote apoptotic processes [35]. However, DOX and the large amount of ROS it generates may upregulate proteins such as Beclin1, Atg7, and Atg12, which leads to excessive autophagy [36]. On the other hand, DOX can activate the tumor suppressor protein p53 and stimulate the Akt/mTOR/ULK1 pathway to inhibit autophagy [37]. Autophagy is a dynamic process. In relevant AIC studies, different DOX dosages, cell or animal strains, genders, treatment durations and methods, and measurements of autophagy markers (p62/SQSTM1, LC3, and Atg) at different times can yield divergent results [33,38,39,40]. Despite the conflicting results regarding whether ANTs inhibit or enhance autophagy, from the perspective of the dynamic characteristics of autophagy, ANTs can impair the autophagic flux [41]. Therefore, defining the impact of autophagy on AIC solely by measuring autophagy-related protein levels without considering changes in autophagic flux lacks persuasiveness [36].

In normal cardiomyocytes, calcium ions are stored and converted within the sarcoplasmic reticulum (SR), mitochondria, and cell membrane. Studies have reported that •O_2_^−^ induced by DOX can lead to calcium ion imbalance through the oxidation of the sulfhydryl sites of ryanodine receptors (RyRs), leading to massive calcium release from the SR [42]. Moreover, doxorubicinol increases the activity of L-type calcium channels by inhibiting sodium–calcium channels, thus increasing the intracellular calcium ion concentration. It can also activate the mPTP, causing a calcium imbalance that damages the contractile function of cardiomyocytes [43,44].

#### 2.2.2. ANTs Induce Cardiomyocyte Death

ANTs can induce cardiomyocyte death through multiple pathways. Pyroptosis is a type of programed cell death that relies on the inflammasome. DOX can upregulate the expression of NLRP3 and induce the assembly and activation of the inflammasome (NLRP3–ASC–caspase-1 complex) to trigger pyroptosis [45]. In addition, DOX affects the caspase-3/GSDME pathway to induce pyroptosis in cardiomyocytes [46]. Ferroptosis is a type of cell death mediated by iron-catalyzed lipid peroxidation. DOX and its metabolites reportedly inactivate the IRP1 iron–sulfur clusters by binding to their iron sites, increasing Fe^3+^ and generating •O_2_^−^. These changes subsequently affect the expression of TfR, FTH, and other related factors, leading to an imbalance in the levels of intracellular iron and aggravating the production of harmful substances such as ROS [47,48]. Apoptosis refers to the programed cell death process triggered by excessive cell damage and is executed mainly through intrinsic and extrinsic pathways. ANTs disrupt the mitochondrial structure and induce the opening of the mPTP through the production of large amounts of ROS, thereby initiating the intrinsic pathway of apoptosis in cardiomyocytes [49]. Moreover, ANTs can upregulate the expression of Fas/FasL and TNF-α, activating the caspase family and thereby triggering the extrinsic apoptotic cascade [50,51].

#### 2.2.3. DNA TOP 2β-Mediated Cardiotoxicity

DNA TOP 2 are the targets of ANTs. There are two types of DNA TOP 2 in mammals: TOP 2α, which is highly expressed in rapidly accreting or dividing cells, and TOP 2β, which is present in all cells [52,53]. DNA TOP 2β is highly expressed in neurons, cardiomyocytes, and other cell types. Upon the administration of anthracyclines (ANTs), this nonspecific and nontargeted anticancer drug forms a ternary TOP 2β-ANT-DNA complex, which breaks the DNA double strand, leading to cardiomyocyte death. This ultimately induces a series of cardiotoxic effects, including arrhythmia, heart failure (HF), and cardiac hypertrophy [4,54]. Zhang et al. [55] constructed a mouse model with a cardiomyocyte-specific deletion of DNA TOP 2β. After DOX treatment, the upregulation of apoptosis-related genes (Trp53inp1, Apaf1, Bax, Mdm2, Fas, etc.) in the cardiomyocytes in this mouse model disappeared, unlike those in the cardiomyocytes of normal mice. Moreover, the number of DNA double strand breaks and the degree of cell apoptosis in the cardiomyocytes of the model mice were significantly reduced.

### 2.3. Clinical Management of AIC

Currently, the treatment of AIC involves a combination of close monitoring and prevention, and the main monitoring methods are imaging surveillance and the detection of serum-related biomarkers [56]. Imaging monitoring tools (e.g., echocardiography) allow for monitoring parameters such as the left ventricular ejection fraction (LVEF), left ventricular global longitudinal strain (LVGLS), and cardiac diastolic function in patients on medication [57]. Although the left ventricular ejection fraction (LVEF) is used as the primary indicator when monitoring patients receiving ANT therapy, it has high temporal specificity and is influenced by the physiological status of the patient. Additionally, its low sensitivity to subtle changes in myocardial contractile function limits its ability to promptly detect subclinical left ventricular dysfunction during monitoring [58]. Compared with the LVEF, the LVGLS is more sensitive to early myocardial injury, and cardiac diastolic function can also indicate abnormalities earlier. However, both of the above methods have to be used in combination with other monitoring methods and myocardial injury indicators [59].

Cardiac troponin (cTn) and brain natriuretic peptide (BNP) are the primary serum-related markers of cardiotoxicity [60]. Changes in cTn provide greater sensitivity when monitoring cardiac function in the early stage of ANT chemotherapy, whereas changes in BNP are more suitable for monitoring chronic or delayed AIC [61]. However, some studies have shown that the predictive role of cTn in cardiotoxicity disappears after ANT treatment [62]. Currently, the commercial detection of cTn may vary due to differences in the sources and platforms of detection reagents, which leads to inconsistent definitions of cTn concentration thresholds, warranting further exploration and research [63]. BNP is a crucial cardiac neurohormone that is synthesized and secreted primarily by ventricular myocytes. BNP has functions such as natriuresis, diuresis, and vasodilation. Additionally, during BNP production, inactive N-terminal pro-B-type natriuretic peptide (NT-proBNP) is released into the bloodstream [64]. BNP is stated as the only class 1A biomarker for measuring HF in the HF guidelines promulgated by the ESC and the American College of Cardiology/American Heart Association (ACC/AHA) [63,65]. In addition to these two classical biomarkers, several novel biomarkers, such as myeloperoxidase, high-sensitivity C-reactive protein, placental growth factor, arginine–nitric oxide metabolites, and topoisomerase 2β, have been validated through clinical studies in recent years [63,66,67,68].

Currently, the only targeted drug used clinically for the treatment of AIC is DRZ, which is an iron chelator that inhibits ferroptosis caused by ANTs. Studies have shown that DRZ can competitively bind to DNA TOP 2β with ATNs to alleviate AIC [69]. In addition, neurohormonal drugs such as ACEIs, ARBs, and β-blockers are available, which, when used in combination with enalapril or carvedilol, can reduce the abnormally elevated LVEF caused by ATNs during the treatment of acute leukemia [70]. ARBs, such as candesartan, may alleviate abnormal left ventricular end-diastolic volume and LVGLS. Beta-blockers, such as carvedilol, may relieve the cTn elevation and diastolic dysfunction caused by ANTs [71]. In addition, lipid-regulating statins, such as atorvastatin, can reduce the LVEF in patients [72]. However, these preventive and curative drugs cause certain toxic side effects, and the studies on these drugs have reported limitations in terms of clinical research sample size and individualized medication [73,74]. Therefore, further exploration is needed to identify safer and universal drugs for AIC treatment.

## 3. Classification of Ginsenosides and Their Therapeutic Effects on Cardiovascular Diseases

### 3.1. Classification of Ginsenosides

**Figure 2:**
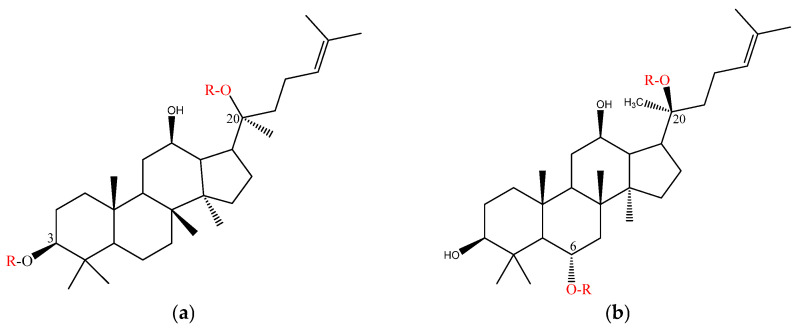

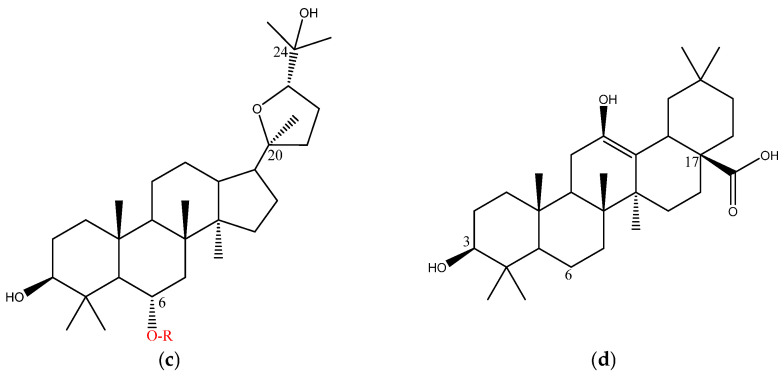
Chemical structures of ginsenosides. (**a**) Protopanaxadiol (PPD); (**b**) protopanaxatriol (PPT); (**c**) ocotillol type; (PPD); (**d**) oleanolic acid type.

Ginsenosides are derived primarily from plants of the *genus Panax* in the Araliaceae family, such as *Panax ginseng*, *Panax quinquefolius*, and *Panax notoginseng*. Ginsenosides are classified into three categories according to their aglycone structure: dammarane type, oleanolic acid type, and ocotillol type [75]. Dammarane-type saponins are further classified into protopanaxadiol (PPD) and protopanaxatriol (PPT) types based on the number of hydroxyl groups, and both are tetracyclic triterpenoid saponins [76]. The oleanolic acid type is a type of pentacyclic triterpenoid saponin that is less common and includes Ro, Ro1, Ri, etc. [75,77]. Ocotillol-type saponins are classified into four configurations, namely, (20S, 24S), (20R, 24R), (20S, 24R), and (20R, 24S), on the basis of the different substituents connected to the C20/C24 positions of the aglycone [78]. These include Makonoside-Rs in *Panax vietnamensis*. Additionally, there are ginsenoside R2, notoginsenoside R1, and pseudoginsenoside F11, which possess a five-membered epoxy ring at the C20 position [79] (Figure 2, Table 1).

### 3.2. Ginsenosides Used to Treat Cardiovascular Diseases

Most ginsenosides possess certain antioxidant, anti-inflammatory, antitumor, and antibacterial activities [80]. Therefore, they can be used to treat obesity, central nervous system diseases, diabetes, and especially various CVDs caused by multiple risk factors [81]. Numerous studies have shown that ginsenosides can be used in the treatment of different CVDs owing to their ability to reduce oxidative stress. The inactivation of the NO signaling pathway mediates endothelial dysfunction, which is a major factor leading to MI and HF. Ginsenosides reportedly reduce vascular abnormalities by restoring NOS function [82]. Additionally, studies have demonstrated that ginsenosides exert therapeutic effects by directly influencing factors related to MI and HF [83]. Oxidative stress can also trigger inflammation, thereby promoting the oxidation of lipids in the vascular endothelium, generating plaques, and thus mediating cardiovascular atherosclerosis [84]. Ginsenosides can decelerate disease progression through multiple pathways [85]. At the level of drug-related complications, ginsenosides exhibit a wide range of therapeutic effects against the cardiotoxic side effects caused by the medications used for disease treatment. Ginsenosides reportedly alleviate the cardiac abnormalities caused by antiarrhythmic drugs affecting ion channels [86]. Additionally, ginsenosides can mitigate the cardiac damage induced by antipsychotics by improving cardiac function and regulating neurotransmitter levels [87]. Finally, various classes of ginsenosides can be used to prevent and control the cardiotoxicity induced by chemical and anti-infective drugs by modulating oxidative stress, inhibiting apoptosis, regulating ion channels, and improving vascular function [88]. The following section of this review elaborates on the therapeutic effects of ginsenosides on AIC from the perspective of AIC pathogenesis.

## 4. Therapeutic Effects and Mechanisms of Ginsenosides in AIC

### 4.1. Ginsenosides Inhibit Oxidative Stress

Oxidative stress is one of the important mechanisms that causes AIC, and the interactions among various regulatory factors and signaling pathways constitute a complex signaling network. Therefore, the process through which ginsenosides alleviate AIC by regulating oxidative stress is also complicated. Generally, ginsenosides alleviate DOX-induced cardiac damage by reducing the levels of ROS and regulating the key nodes of oxidative stress through the modulation of various signaling factors and pathways. For example, Xie et al. [89] reported that ginsenoside Re attenuated oxidative injury in cardiomyocytes induced by exogenous H_2_O_2_ and reduced endogenous ROS production triggered by DOX. Wang et al. [90] reported that DOX decreased myocardial SOD, GSH, and CAT levels while increasing serum CK, LDH, and AST levels in mice. These indices normalized after ginsenoside Rh2 treatment, whereas improvements in myocardial tissue sections and electrocardiograms in mice were observed, demonstrating that ginsenoside Rh2 can be used as a cardioprotective agent against AIC. Maintaining a balanced intracellular calcium concentration is important to ensure the specialized physiological activities of cardiomyocytes. Li et al. [91] demonstrated through in vivo and in vitro experiments that the micelle-encapsulated ginsenoside Rg3 (p-Rg3), in addition to enhancing the antitumor efficacy of DOX, mitigates AIC by inhibiting intracellular calcium overload in cardiomyocytes. This effect may be associated with the phenomenon in which p-Rg3 restores sarcoplasmic reticulum imbalance caused by DOX.

**Figure 3:**
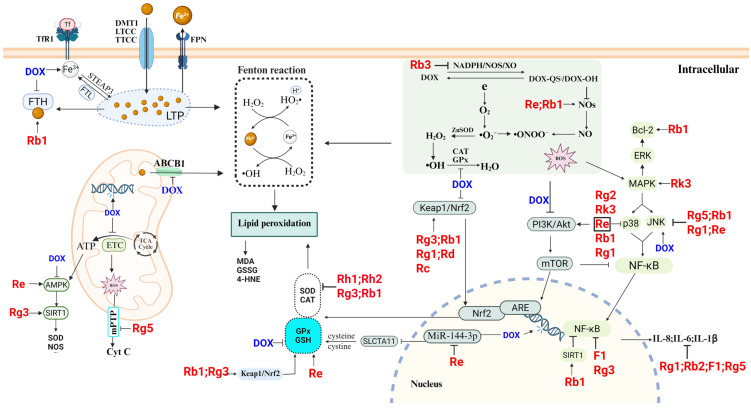
The mechanism by which ANTs induce AIC through oxidative stress is rather complex. In this regard, ginsenosides exert protective effects by antagonizing oxidative stress, inflammation, and ferroptosis via multiple targets. DOX inhibits the activation of the Keap1/Nrf2 pathway, blocking Nrf2 nuclear translocation, thereby downregulating the expression of antioxidant genes such as SOD, GSH, and CAT. However, ginsenosides Rg3 [91], Rb1 [116], etc., can reverse this process. Meanwhile, ginsenosides Rg2 [114] and Rk3 [103], etc., counteract the inhibitory effects of DOX on the PI3K/Akt/mTOR pathway, thus exerting antioxidant effects. Furthermore, ginsenosides can alleviate inflammation and ferroptosis induced by DOX and ROS through regulating upstream and downstream signaling molecules.

Increasing the antioxidant capacity is the main means of preventing and treating AIC. Wang et al. [92] reported that ginsenoside Rg3-DOX cotreatment could alleviate the abnormal vasoconstriction and diastole caused by DOX. This effect was related to the fact that ginsenoside Rg3 reduced eNOS in a dose-dependent manner and downregulated ET-1 release through a negative feedback mechanism. Additionally, this study revealed that ginsenoside Rg3 can alleviate calcium disorders, significantly increase SOD activity, and restore the balance of SOD-1/GPx and SOD-2/GPx. These results suggest that ginsenoside Rg3 counteracts DOX-induced cardiac injury by activating the Nrf2/ARE and AKT signaling pathways (Figure 3). Similar findings were reported in another study on pirarubicin (THP)-induced AIC. Ginsenoside F1 was reported to reduce the increases in MDA, cTn, and LDH in the serum of THP-induced mice, increase the activity of antioxidant components such as SOD and GSH, and improve their antioxidant capacity. Ginsenoside F1 reportedly alleviated THP-induced cardiotoxicity by promoting the nuclear translocation of Nrf2 and regulating the Keap1/Nrf2/ARE pathway to mitigate oxidative stress [93]. Alleviating the DOX-induced inflammation caused by excessive ROS, in addition to reducing myocardial pathological damage, prevents the exacerbation of oxidative stress. As mentioned above, Wang et al. [92] reported that Rg3 inhibits the levels of inflammatory factors such as VEGF and TGF-β to reduce the inflammatory response of the heart to DOX. In addition, Zhang K et al. [94] constructed an AIC model through an intraperitoneal injection of DOX in mice. The authors reported that Shenmai Yin (SMY) preparation containing ginsenoside Rg1 reduced the levels of TNF-α and IL-6 in a dose-dependent manner, decreased the levels of the ventricular remodeling markers MMP-2 and MMP-9. Moreover, ginsenoside Rg1 also reduces extracellular matrix (ECM) deposition by inhibiting the myocardial fibrosis factor COL-IV. These findings indicate that SMY can mitigate the risk of myocardial fibrosis and cardiac remodeling induced by ANTs. Hou J et al. [95] reported that ginsenoside Rh2 regulates p21 to reduce the protein expression levels of the fibrosis markers α-SMA and COL1A1 and alleviates DOX-induced myocardial fibrosis and inflammation by reducing the release of IL-1A, IL-18, and TNF. The results of the apoptosis array analysis conducted in this study revealed that the ginsenoside Rh2 prevents DOX-mediated cardiomyocyte death by inhibiting both the intrinsic and extrinsic apoptotic pathways. This was demonstrated mainly by downregulating the high expression of the proapoptotic proteins Toll-like receptor (TLR) and TRAIL-R2 and inhibiting the activity of factors such as CAT and HO-2. Ginsenosides alleviate DOX-induced cardiotoxicity by modulating oxidative-stress-related mechanisms, as shown in Figure 3.

Inhibiting oxidative stress is pivotal in the treatment of AIC. In addition to being a major inducer of AIC, ROS imbalance is an underlying mechanism and developmental process in various cardiovascular diseases. In a study on a myocardial ischemia/reperfusion (MI/R) injury model, ginsenoside Rg3 modulated Akt/eNOS signaling and improved cardiomyocyte oxidation levels, resulting in the alleviation of MI in mice [96]. Jia Y et al. [97] conducted network pharmacology experiments and demonstrated that ginsenoside Rg3 reversed the isoproterenol-induced increases in IL-6 and TNF-α levels, thereby alleviating the inflammatory response. These findings indicate that the targets and mechanisms of ginsenosides in the treatment of AIC overlap with those in other cardiovascular diseases. Therefore, it is understood that the mechanisms through which ginsenosides treat other types of CVDs may provide potential targets for the prevention and treatment of AIC (Table 2 and Table 3; Figure 3).

**Table 2:** The treatment mechanism of ginsenosides for oxidative stress in other types of cardiovascular diseases.

Ginsenosides	Models	Disease	Mechanism	Reference
Rg3	Angiotensin II (Ang II)-induced cardiac hypertrophy in vitro; in vivo transverse aortic constriction constructs a rat model of cardiac hypertrophy.	Hypertensive Cardiac Hypertrophy (HCH); Myocarditis (MC)	ANP, BNP, and β-MHC ↓; myocardial fibrosis-related proteins (MyHc, CollagenI, and TGF-β1) ↓; upregulation of Nrf2/HO-1, SIRT1 pathway; downregulation of NF-κB pathway; Regulation of Nrf2/HO-1, SIRT1/NF-κB pathway inhibits inflammation and oxidative stress.	[98]
Rg5	Ang II induces cardiac inflammation and remodeling.		IL-1β, IL-16, TNF ↓; p-JNK ↓; AP-1 ↓; Inhibition of the JNK/AP-1 pathway blocks inflammation.	[99]
Rb3	Ang II injury to cardiomyocytes.		NADPH ↓; ROS ↓; NOX-2, NOX-4, p67 ↓; NO, NOS ↑; Reversing NADPH overexpression to resist oxidative damage.	[82]
F1	A mouse model of atherosclerosis constructed by feeding a high-fat diet.	Atherosclerosis	LOX-1, TLR4 ↓; MPO ↓; G-CSF, ICAM-1, MIP-1δ, IL-1α, IL-15, IL-16 ↓; A20, NF-κB ↓; Mediated A20 inhibition of the NF-κB signaling pathway relieves inflammation.	[100]

↓: Decrease; ↑: Increase.

**Table 3:** The mechanisms by which ginsenosides alleviate MI, HF, and ICM through oxidative stress.

Ginsenosides	Models	Disease	Mechanism	Reference
Rg1 Rd Rk3 Rh1 Rb1 Rd Rc	In vitro cultivation of H9c2 cardiomyocytes subjected to hypoxia/reoxygenation (H/R) injury	MI, HF, Ischemic Cardiomyopathy (ICM)	SOD, GSH-Px, GSH ↑; LDH ↑; Nrf2, HO-1 ↑; TNF-α ↓; Activates Nrf2/HO-1 signaling pathway, inhibits JNK phosphorylation, and regulates Akt and MAPK pathways to prevent oxidative stress	[101,102,103,104,105,106,107]
Rg1	In vivo and in vitro ischemia/reperfusion (I/R) injury models		Protection of cardiomyocytes against hypoxia-induced cellular injury by upregulation of HIF-1α through activation of the PI3K/Akt/mTOR pathway	[108]
Rb1 Re	Rat myocardial I/R model: H_2_O_2_-induced oxidative stress		CK, MDA ↓; LDH ↑; SOD, eNOS, NO ↑; increase NO content to inhibit oxidative stress	[109,110]
Rb1	In vitro H/R model of H9c2 cardiomyocytes		SOD, CAT, GSH-Px ↑; MDA ↓; PARP-1/2 ↑; ERα, ERβ, p-Akt ↑; p-JNK, p-ERK 1/2 ↓; Activation of ER-dependent crosstalk across Akt, JNK, and ERK 1/2 pathways prevents H/R damage	[111]
Rg1	In vitro H/R model of H9c2 cardiomyocytes		CK, LDH ↓; MMP, ATP ↑; Bax/Bcl-2, ROS ↓; GDH ↓; MFN2 ↑; Regulates GDH and MFN2, maintains mitochondrial dynamics to prevent H/R injury	[112]
Rg5	Modeling myocardial ischemia		Promotes Akt translocation, increases mitochondrial hexokinase-II (HK-II) binding to mitochondria; inhibits dynamin-related protein 1 (Drp1) recruitment and mitochondrial fission; mPTP ↓; ATP ↑; Regulation of HK-II and Drp1 increases resistance to hypoxia/reoxygenation injury	[113]
Rg2	Ang II injury to cardiomyocytes	MI	Col-1, Col-3, α-SMA ↓; p-Akt ↑; Masson staining shows Rg2 reduces MI-induced cardiac fibrosis in mice	[114]
Re	Constructing a rat MI model		P-AMPKα ↑; TGF-β1 ↓; Smad2/3 ↓; FAK, p-PI3K/p110α, p-Akt ↑; Regulation of AMPK/TGF-β1/Smad2/3 and FAK/PI3K p110α/Akt signaling pathways	[115]

↓: Decrease; ↑: Increase.

### 4.2. Inhibition of Ferroptosis by Ginsenosides

Numerous studies have demonstrated that ANTs induce ferroptosis in cardiac cells and thus aggravate AIC. Research on ginsenosides has confirmed their inhibitory effects on ferroptosis. In related mechanistic studies, ginsenosides alleviate DOX-induced myocardial injury by regulating the Nrf2 pathway and thus inhibiting ferroptosis. Zhai et al. [116] established an acute DOX cardiotoxicity model and reported decreased expression of the ferroptosis protein markers FTH1 and GPX4. Following treatment with ginsenoside Rb1, the expression of FTH1 and GPX4 increased, whereas Nrf2 was activated and translocated to the nucleus, leading to increased expression of antioxidant genes. In another study on cardiac injury, similar conclusions were drawn. This study revealed that ginsenoside Rg3 mitigated ferroptosis in I/R-induced model mice by regulating the Nrf2 signaling pathway. Additionally, studies using in vitro models have demonstrated that ginsenoside Rg3 activates the keap1/Nrf2 pathway to inhibit hypoxia-induced ferroptosis [117]. Furthermore, miR-144-3p was revealed as a key gene in the induction of ferroptosis in another study using an MI/R injury model, and its target gene, SLC7A11, was found to be downregulated upon MI/R injury due to miR-144-3p overexpression, thereby triggering ferroptosis [118]. Ye J et al. [118] reported that SLC7A11 expression was increased and attenuated cardiac injury after treatment with ginsenoside Re. The evidence suggests that ginsenoside Re can alleviate myocardial I/R injury by attenuating ferroptosis through the miR-144-3p/SLC7A11 pathway. Notably, although ANTs do not directly induce I/R injury, ferroptosis triggers endoplasmic reticulum stress, which is closely linked to I/R injury. Therefore, the mechanism of ginsenosides in the treatment of AIC through the inhibition of ferroptosis may represent a critical therapeutic target worth studying (Figure 3).

### 4.3. Regulation of Autophagy by Ginseng Saponins

Autophagy is an important mechanism of ANT-induced cardiotoxicity and, notably, many studies have shown that ginsenosides can regulate autophagy to alleviate AIC through multiple pathways. Autophagy is primarily divided into four stages: the initiation of autophagy mediated by the ULK1 complex and the Beclin-1 complex; the extension of the phagophore and the capture of materials to be degraded promoted by the conversion of LC3-I and the Atg5–Atg12–Atg16L complex; the fusion of the mature autophagosome and lysosome to form the autolysosome; and, finally, the degradation of the contents of the autophagosome by lysosomal enzymes, which releases harmful substances and metabolites [41,119,120]. Under normal circumstances, p62 and LC3-II serve as autophagy markers. An increase in the LC3-II/LC3-I ratio indicates increased autophagy. A decrease in the protein level of p62 protein, which serves as an autophagy substrate, indicates that the autophagy degradation process is promoted, whereas the opposite indicates abnormal autophagy degradation and impaired autophagic flux [121]. Notably, multiple reports have indicated the dualistic effects of ginsenosides on autophagy. On the one hand, ginsenosides can inhibit autophagy. For example, Zhai Y et al. [116] reported in their study on the alleviation of DOX-induced AIC by ginsenoside Rb1 that DOX could activate autophagy by phosphorylating the AMPK/ULK pathway and inhibiting mTOR. However, after treatment with ginsenoside Rb1, downregulation of the AMPK pathway alters the DOX-induced upregulation of p62 and LC3-II, thereby regulating autophagy and reducing toxicity [122]. Ginsenoside Rg1 can also downregulate the expression of proteins such as p62, LC3-II, and Beclin-1 through this mechanism [123]. Gao C et al. [124] also found that ginsenoside Re reduces the protein expression of LC3-II and p62 protein levels by inhibiting the Ras/MEK/ERK1/2 signaling pathway. On the other hand, ginsenosides can promote autophagy. As a positive regulator of the initiation phase of autophagy, the phosphorylation of AMPK is inhibited by ANTs to prevent autophagy initiation [41]. Qiao L et al. [125] reported that ginsenoside Rb1 mediated AMPK phosphorylation, increased LC3II levels, and promoted SQSTM1/p62 degradation to restore autophagic flux and, thus, alleviate atherosclerotic disease. In a study on acute myocardial ischemia, ginsenoside Rb1 was shown to regulate key proteins in the PINK1/Parkin pathway, such as increasing the expression of PINK1, Parkin, and LC3-II/LC3-I to promote the initiation of autophagy; decreasing the expression level of p62 to increase degradation; and combining with the FUNDC1 pathway, thus promoting mitochondrial autophagy [126]. The same conclusion was reached when ginsenosides Rg3 and Rg1 were used to prevent cardiac remodeling caused by HF [127,128]. Zhang HY et al. [129] reported that ginsenoside Rg1 can alleviate hypoxia/reoxygenation (H/R) injury in HL-1 cardiomyocytes by inhibiting autophagy through the miR-155/Notch1/Hes1 pathway.

**Figure 4:**
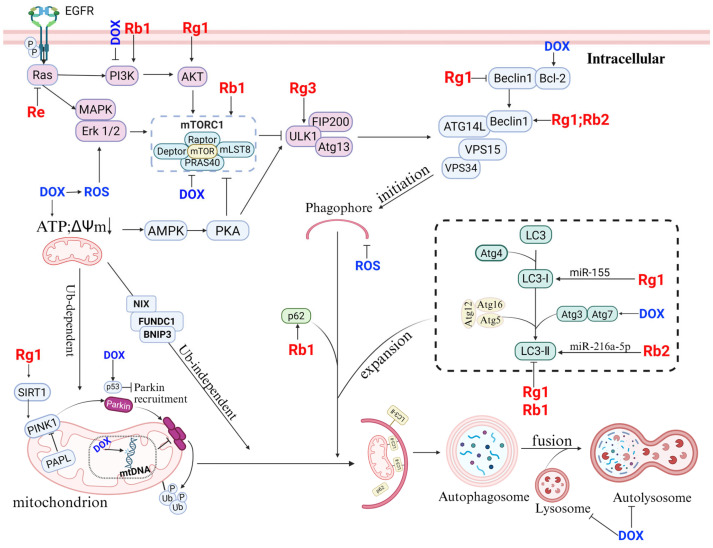
Ginsenosides maintain myocardial homeostasis through bidirectional regulation of autophagy. On the one hand, ginsenosides such as Rb2 [130] and Rg1 [135] promote mitochondrion autophagy by activating the SIRT1/PINK1/Parkin pathway and regulating key factors such as Beclin1 and p62, thereby clearing damaged mitochondrion. On the other hand, by regulating pathways and targets such as the PI3K/Akt/mTOR signaling axis and miR-216a-5p/LC3-II, ginsenosides inhibit DOX-induced excessive autophagy and alleviate AIC.

In addition to proteins such as LC3 and p62, Beclin 1 and Atg5 regulate autophagy. Studies have shown that ginsenoside Rb2 can promote autophagy to treatment for HF by downregulating miR-216a-5p to increase the expression of signaling molecules such as LC3B II/I and Beclin1 [130]. Ginsenoside Rg1 can dissociate the Bcl-2–Beclin 1 complex to initiate autophagy and clear damaged substances, thereby protecting cardiomyocytes [131]. PI3K/Akt and MAPK are two key signaling pathways in autophagy, and the three main components of the MAPK signaling cascade are ERK, JNK, and p38 [103]. The activation of Akt, as well as the phosphorylation of MAPK, can alleviate DOX-induced cardiac injury [132]. Zhu C et al. [133] reported that ginsenosides can increase the phosphorylation of Akt and ERK. Qin GW et al. [134] discovered that ginsenoside Rb1 regulates the PI3K/Akt/mTOR pathway, inhibits autophagy, and alleviates ischemia/reperfusion (I/R) injury. Thus, activation of the Akt and Erk pathways could be one of the mechanisms through which ginsenoside Rb1 alleviates DOX cardiotoxicity. In summary, ANTs’ dual regulation of autophagy results in cardiotoxicity and has different regulatory effects as the disease progresses (Figure 4). In the early stages of disease, appropriate autophagy can serve as a compensatory mechanism to clear harmful substances. However, DOX increases the mRNA levels of proteins such as Beclin1 and Atg7 during the initiation of autophagy, resulting in excessive autophagy that exacerbates AIC. In later stages, DOX has a negative impact on the quantity and function of lysosomes, as does the subsequent fusion of autophagosomes with lysosomes, thereby blocking autophagic flux and leading to the accumulation of harmful substances. Therefore, the role of ANT-mediated autophagy in AIC requires exploration from multiple angles and levels. However, many studies that judge whether autophagy can alleviate AIC based solely on the regulatory effects of ginsenosides on autophagy-related proteins such as p62 and LC3 are not comprehensive (Table 4). A comprehensive and integrated analysis of the dynamic process of autophagy is needed. Therefore, in subsequent studies, it may be worthwhile to focus on exploring the regulatory effects of ginsenosides on the autophagy flux.

### 4.4. Inhibitory Effects of Ginsenosides on Pyroptosis

Ginsenosides exhibit anti-inflammatory effects, and NLRP3 inflammasome activation, a key node triggering inflammation, also serves as a critical protein complex for pyroptosis initiation. Numerous studies have confirmed that ginsenosides regulate NLRP3 through multiple pathways, thereby playing a role in inhibiting pyroptosis [136]. First, ginsenosides can alleviate MI. Bing Li et al. [137] demonstrated, by constructing an ex vivo MI model, that the ginsenoside Rh2 could prevent the activation of NLRP3 inflammatory vesicles. Second, ginsenoside Rh2 inhibits the expression of the pyroptosis marker protein GSDMD and other key proteins, such as caspase-1 and ASC, and also reduces the release of inflammatory factors such as pro-IL-1β and pro-IL-18. In summary, this study demonstrated that ginsenosides alleviate MI by inhibiting NLRP3-mediated pyroptosis. Another study using H/R models to simulate MI/R injury revealed that SIRT1 mediates the deacetylation of ASC, thereby influencing inflammasome formation. Rb2 attenuates ASC acetylation by upregulating SIRT1 expression, which, in turn, inhibits the activation of NLRP3 inflammatory vesicles to alleviate MI/R [138].

**Figure 5:**
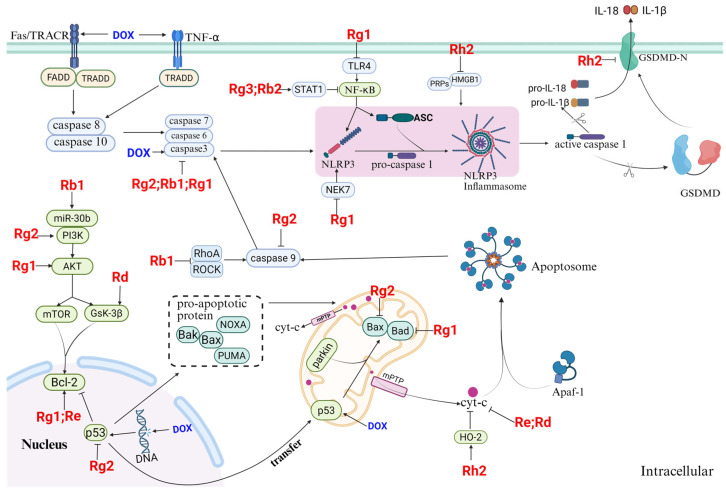
Mechanism of ginsenoside inhibition of cellular pyroptosis and apoptosis to alleviate AIC. DOX activates factors such as Fas/FasL and TNF-α to trigger extrinsic apoptosis and promote ROS production, antagonizing DOX/ROS-induced apoptosis. In contrast, ginsenosides can inhibit pro-apoptotic signals such as the caspase family and p53 and upregulate the anti-apoptotic protein Bcl-2, thereby antagonizing DOX-induced or ROS-induced apoptosis. Additionally, regarding the intrinsic apoptosis pathway, ginsenosides inhibit the release of cyt-c, reduce the activity of pro-apoptotic proteins such as Bax and Bad, and thereby prevent the formation of apoptosome. In the pyroptosis process mediated by the NLRP3 inflammasome, ginsenosides such as Rg3 [98] and Rg1 [142] play a key role by inhibiting the formation of the NLRP3 inflammasome.

Qi Z et al. [139] conducted a study on the mechanism of the Chinese patent medicine Qishen Yiqi Pill (QSYQ) in the treatment of MI. They reported that ginsenoside Rh2, a crucial component of QSYQ, could bind to high-mobility group box 1 (HMGB1), thereby preventing HMGB1 from interacting with pattern recognition receptors (PRRs) to activate the NLRP3 inflammasome, resulting in the inhibition of the cleavage of the pyroptosis execution protein GSDMD. Ginsenosides can also inhibit the NLRP3 inflammasome and oxidative stress induced by Ang II. Mechanistically, this is achieved mainly by upregulating SIRT1 in cardiomyocytes to inhibit the NF-κB pathway, thereby preventing the expression of the NLRP3 inflammasome and oxidative stress markers [98]. In addition, studies employing TLR4 siRNA, p-NF-κB inhibitors, and NLRP3 siRNA revealed that ginsenosides attenuate cardiomyocyte apoptosis and inflammation by blocking the TLR4/NF-κB/NLRP3 pathway [140]. Notably, the formation of the NLRP3 inflammasome initially requires the binding of NLRP3 to NEK7, which subsequently induces the formation of the NLRP3 inflammasome platform and promotes the filamentation of the NLRP3 PYD domain [141]. As discussed previously, the inhibitory effect of ginsenosides on the formation of the NLRP3 inflammasome may be due to the suppression of caspase-1 and ASC expression. Recently, a study demonstrated that ginsenosides may also inhibit the binding of NLRP3 to ASC, the oligomerization of ASC, and the formation of pyroptosis spots through the disruption of the NEK7–NLRP3 interaction [142]. Ginsenosides can play a therapeutic role in diseases such as HF and myocardial infarction (MI) by regulating the NLRP3 inflammasome. Although few studies have shown that ginsenosides alleviate AIC by inhibiting pyroptosis, NLRP3 is a key protein in pyroptosis, and DOX can modulate the process of NLRP3 via pathways such as the ROS or NF-κB. Consequently, further investigation into the potential of ginsenosides to attenuate AIC by targeting NLRP3-mediated pyroptosis holds considerable scientific and therapeutic value (Figure 5).

### 4.5. Inhibition of Apoptosis by Ginsenosides

The mechanisms through which DOX induces cardiac cell apoptosis are relatively common. On the one hand, ginsenosides may directly regulate the expression of apoptotic proteins such as Bcl-2, HO-1, Bax, XIAP, caspase, and Ki67 to achieve cardioprotective effects. Studies have demonstrated that the combined use of *ginseng* and febuxostat significantly reduces the DOX-induced increases in cTn and BNP and downregulates caspase-3 to inhibit cardiomyocyte apoptosis [143]. Hou J et al. [95] confirmed through apoptosis chip data that ginsenoside Rh2 increases the expression of proteins such as HO-2, HSP27, and XIAP, which are downregulated by DOX. This study demonstrated that the ginsenoside Rh2 inhibits both intrinsic and extrinsic apoptosis pathways, thereby preventing cardiomyocyte apoptosis. On the other hand, ginsenosides may alleviate AIC by regulating the upstream signaling pathways of various apoptosis-related proteins. Peng et al. [144] established a DOX-induced chronic heart failure (CHF) model and demonstrated that ginsenoside Rg1 increased the phosphorylation levels of Akt and Erk. This modulation resulted in altered expression of the downstream signaling molecules mTOR, Bcl-2, and Bad, thereby inhibiting the protein expression of caspase3 and Bax, while upregulating the anti-apoptotic gene Bcl-2 to counteract DOX-induced CHF. Activation of PI3K/Akt pathway phosphorylation maintains the anti-apoptotic function of Bcl-2 and inhibits the activity of caspase-9. In addition, the upregulation of p53 activates the expression of pro-apoptotic genes such as Bax and PUMA, thus eliminating the cells with excessive DNA damage through the mitochondrial endogenous apoptosis pathway. Studies have demonstrated that ginsenoside Rg2 attenuates DOX-induced cardiomyocyte apoptosis, possibly by downregulating the expression of the p53 gene and increasing PI3K/Akt pathway phosphorylation [145]. Rb1 also exerts similar effects. The upregulation of the miR-130b-mediated PTEN/PI3K/AKT signaling pathway reduces the expression of apoptosis-related factors such as caspase3 and Ki67 in cardiomyocytes, thereby inhibiting apoptosis and alleviating AIC [146].

Aromatic hydrocarbon receptor (AhR) is a ligand-dependent transcription factor. The study by Zhang Y et al. [147] reported that ginsenoside Rb1 can act as an AhR agonist and binds competitively to AhR with DOX, reducing the expression of the downstream target gene CYP1A associated with apoptosis and thereby exerting a protective effect against AIC. Reducing the accumulation of DOX in cardiomyocytes is crucial for preventing the occurrence of AIC [148,149]. Modifying the dosage form of DOX to decrease drug uptake by the heart can be used as a treatment approach to alleviate AIC. Li C et al. [150] developed a codelivery system of nanoparticles by self-assembling Rg1 micelles to encapsulate DOX. Dox@Rg1 nanoparticles can reduce the deposition of DOX in cardiac cells and tissues and downregulate p53 and caspase-3, thereby alleviating AIC. In other type of CVDs, ginsenosides also relieve diseases such as HF and MI by regulating the same targets. For example, the combined use of ginsenosides Rb3 and Rb2 can increase the expression of Bcl-2, downregulate Bax and caspase-3, and reduce MI/R injury [151]. In addition, other studies have demonstrated that ginsenosides Re, Rb1, and Rd can influence relevant apoptotic genes (Bcl-2/Bax, caspase-9, and caspase-3) by regulating certain signaling pathways, such as the Nrf2/HO-1/PGC-1α pathway, the RhoA/ROCK signaling pathway, and the Akt/GSK-3β signaling pathway [111,152,153]. These effects enable ginsenosides to treat myocardial diseases induced by triggers such as MI/R, oxidative stress, and hypoxic injury. These phenomena indicate a high degree of overlap among the mechanisms through which ginsenosides alleviate AIC by inhibiting apoptosis-related pathways and their mechanisms in treating other cardiovascular diseases. Therefore, the mechanisms of ginsenosides in the treatment of non-AIC-related myocardial diseases may provide novel insights for further expanding prevention and treatment strategies for AIC (Figure 5).

### 4.6. Protective Effects of Other Factors on AIC

In addition to exerting a protective effect by directly regulating the molecular mechanisms of AIC, ginsenosides also modulate changes in lipid levels, angiogenesis, and anticoagulation during the progression of CVDs [88,154]. In recent years, the natural active components of plants can exert a “medicinal enhancement and toxicity reduction” effect on tumor diseases. In addition to ginsenosides, other saponins such as astragaloside IV [155], saikosaponin D [156] and notoginsenosides [157], as well as non-saponin components, such as capsaicin [158], curcumin [159], cinosylvin [160], and resveratrol [161], also exhibit therapeutic effects in AIC. However, these active ingredients focus primarily on antioxidant and anti-inflammatory activities and, compared with ginsenosides, their protective effects on AIC have certain limitations. For example, capsaicin can inhibit DOX-induced myocardial cell apoptosis through the PI3K–Akt signaling pathway [162]. However, high doses of capsaicin may cause nerve damage that could eliminate endogenous cardiac protective effects [163]. The low water solubility and bioavailability of curcumin limit its efficacy [164]. The efficacy of resveratrol is limited by its rapid metabolism [165]. When screening for natural active ingredients to treat AIC, safety should be the primary consideration, followed by an assessment of whether it affects the efficacy of the chemotherapy drugs themselves, as well as other factors such as bioavailability, stability, and clinical application. Although ginsenosides also have certain limitations, they have a significant advantage in combating AIC.

## 5. Clinical Applications of *Ginseng* and Ginsenosides

The clinical applications of *ginseng* and ginsenosides have been extensively explored. They have been shown to alleviate symptoms such as type 2 diabetes [166], hypertension [167], acute ischemic stroke [168], gastrointestinal damage and inflammation [169], fatigue [170], tumors [171], and chronic kidney disease [172]. Additionally, it is often used as an adjunctive drug in surgery and chemotherapy to improve clinical efficacy and reduce the incidence of adverse reactions [173]. Clinical studies on the treatment of CVDs with ginsenosides are continuously being conducted and deepened [16,174] (Table 5). Currently marketed ginsenoside-based drugs are mostly used for the treatment of CHF, intraoperative IR/I injury, coronary heart disease (CAD), and other conditions (Table 6). There are relatively few clinical studies on the treatment of AIC with *Panax ginseng* and ginsenosides. A randomized controlled clinical trial investigated the protective effect of *ginseng* on AIC. The results showed that *ginseng* supplementation could prevent the early decline in LVEF induced by doxorubicin, thereby preventing cardiac dysfunction associated with cancer treatment [175]. Aidi injection, which is rich in ginsenosides, when used in combination with standard chemotherapy, can significantly enhance anticancer clinical efficacy and reduce chemical toxicity [176,177]. Studies have shown that Aidi injection has a significant inhibitory effect on chemotherapy-induced hematotoxicity, bone marrow suppression, and gastrointestinal side effects, but research on chemotherapy-induced cardiotoxicity is relatively scarce [178,179,180]. However, registered *ginseng* clinical trials (R-GCTs) are gradually becoming more common. For example, a recently submitted study on the prevention of AIC using Shengmai San has been registered, but the results have not yet been uploaded. These R-GCTs also provide certain assistance and reference value for the clinical translation of ginsenosides in the treatment of AIC [174].

Notably, the safety of *Panax ginseng* and its active ingredients in clinical applications appears to yield conflicting results according to existing studies. A double-blind, randomized clinical trial was conducted by Zhang L. et al. [181] on the safety and efficacy of Korean red *ginseng* in patients with deficiency syndrome. The four-week trial indicated that Korean red *ginseng* has a significant anti-fatigue effect on people with deficiency syndrome, with no notable adverse reactions. However, another randomized controlled trial involving Korean red *ginseng* reported that, although there were no significant differences in adverse drug reactions (ADRs) between the two groups, participants experienced headaches, diarrhea, and dizziness during the 24-week trial period [182]. Therefore, further exploration and confirmation of the safety of *ginseng* are needed. Lee NH et al. also found that *Panax ginseng* root extract caused mild ADRs such as indigestion, hot flashes, and insomnia in healthy subjects in a 4-week randomized double-blind controlled clinical trial, but no toxic reactions were observed [183]. Second, although there are numerous clinical studies on the treatment of CVDs with *ginseng* and ginsenosides, several limitations still remain, such as small sample sizes, short observation periods, lack of rigorous control group settings, and inconsistent endpoint criteria. In terms of drug interactions, clinical and preclinical studies have demonstrated that the combination of *ginseng* and therapeutic drugs can increase the efficacy and reduce the incidence of side effects. However, there have also been instances where the combination of certain drugs resulted in increased toxicity and reduced efficacy [184,185]. Therefore, in practical clinical applications, it is necessary to conduct research on the basis of specific circumstances and avoid making blanket statements [186]. Additionally, clinical trials have focused primarily on the comprehensive therapeutic effects of *ginseng* total extracts or total saponins on diseases. This also indirectly reflects that the “multitarget, multi-pathway” advantages of ginsenosides may pose certain obstacles during clinical translation [187]. Finally, the low bioavailability of ginsenosides [187], their complex metabolic transformation, and the difficulty in purifying individual saponins are significant challenges that need to be overcome in the study of their therapeutic effects [186].

**Table 5:** Clinical application of *ginseng* and ginsenosides in CVDs treatment.

Ingredients	Disease	Subjects	Outcome	References
*Panax ginseng* extract (PGE)	The effects of *ginseng* extract on blood pressure and heart rate	30 healthy adult subjects. Oral administration of 200 mg of ginseng extract. Monitoring of ECG parameters and blood pressure.	QTc interval prolongation and diastolic blood pressure after 2 h of ingestion.	[181]
Powder composed of Radix Ginseng, Radix Notoginseng, and Succinum	Coronary artery angina	116 patients with coronary artery angina pectoris. Randomized double-blind trial of treatment group and control group (compound Danshen tablets).	The general symptoms, physical strength, ECG parameters, and lipid metabolism in the *ginseng* treatment group were all better than those in the control group.	[188]
*Red ginseng* extract	ST-elevation acute myocardial infarction (AMI)	50 patients with AMI. Measurement of coronary flow reserve (CFR) and changes in absolute numbers of circulating angiogenic cells.	During the 8-month follow-up period, the CFR in the red *ginseng* group was significantly better than that in the placebo group; CD34+, CXCR4+, and CD117+ levels increased and inflammation slowed down.	[189]
PGE	The effect of PGE on lipid metabolism—lipid-lowering research	Eight adult male subjects, 6 g/day, 8 weeks. Testing serum MDA, SOD, CAT, serum total cholesterol (TC), triglyceride (TG), LDL, HDL, and other indicators.	MDA, TC, TG, LDL ↓; HDL, SOD, CAT ↑. Lowering blood lipids and antioxidant properties.	[190]
*Korean red ginseng* (KRG)	Endothelial function	16 healthy participants on four occasions were administered: KRG root (3 g), KRG ginsenosides extract, KRG polysaccharides extract, and cornstarch control. Assessment of flow-mediated vasodilatation.	Maximum vasodilation occurred 180 min after taking KRG ginsenoside extract. Improves endothelial function in healthy individuals.	[191]
Ginsenoside Rg3–enriched *Korean red ginseng* (Rg3-KRG)	Arterial stiffness and peripheral and central BP	23 healthy subjects. 400 mg Rg3-KRG extract or 400 mg wheat bran control. Measurement of aortic augmentation index and central BP.	Brachial systolic and diastolic BP ↓. Lowers central and peripheral arterial pressures in healthy adults.	[192]
*Ginseng* (Rb1/Rg1)	Blood lipid levels.	Patients with metabolic syndrome, healthy volunteers, postmenopausal women. Meta-analysis.	Total cholesterol, LDL, triglycerides ↓. Regulate blood lipid levels.	[154]

↓: Decrease; ↑: Increase.

**Table 6:** Clinical application of marketed drugs for CVDs treatment.

Ingredients	Disease	Subjects	Outcome	References
Shenmai injection (SMI)	CAD and CHF. Efficacy and safety.	240 patients with CHF complicated by CAD. CHF standard treatment drugs and SMI (100 mL/day). 1 week. Endpoints: NYHA functional classification, SF-36. Heart survey score, traditional Chinese medicines syndrome score, LVEF, and BNP level.	Each endpoint is superior to the placebo group. SMI can further improve the course of patients with CHF complicated by CAD.	[193]
SMI	Coronary heart disease (CHD)	40 patients with OPCABG. Injecting SMI before performing OPCABG. Indicators: cardiac output (CO), stroke volume (SV), and the ejection fraction (EF) during surgery.	CO, SV, EF ↑. Improving the safety of anesthesia.	[194]
Shenmai and compound danshen injection (SM-DS)	Myocardial reperfusion injury after percutaneous coronary intervention (PCI) in patients with acute AMI.	38 patients with AMI who underwent PCI treatment. SM-DS therapy was used before and after PCI surgery. The integrated left ventricular ejection isometric index (Tei) was determined by echocardiogram. Monitoring MDA, SOD, IL-6, and TNF-α levels.	SOD ↑; MDA, IL-6, TNF-α ↓; The improvement time of the Tei index in the treatment group was earlier than that in the control group. SM-DS could reduce the myocardial reperfusion injury in patients with AMI after PCI.	[195]
SMI	CHF	64 patients with CHF. Basic treatment and SMI. 14 days. Tissue Doppler imaging (TDI) monitoring of eft ventricular diastolic function (LVDF) used.	TDI assessment shows that SMI could effectively improve the LVDF in CHF patients.	[196]
SMI	AMI	Meta-analysis of 50 clinical studies of Shenmai for AMI.	The incidence of cardiac failure, the incidence of HF, shock, and reinfarction ↓. No serious adverse drug reactions (ADRs)/adverse events (AEs) were observed, but post-marketing safety evaluation is still required.	[197]
Shenfu injection (SFI)	I/RI	40 patients’ mitral valve replacement (MVR) with cardiopulmonary bypass (CPB). Monitoring systolic SBP, HR, MBP, DBP, and CTn, SOD, and MDA.	MDA, cTnl ↓; SOD ↑. The SBP, MBP, and DBP values and HR were significantly improved in group IV compared with any other groups.	[198]
Xinyue capsule	CAD	A randomized, double-blind, controlled clinical trial involving 1054 CAD patients undergoing PCI. Conventional treatment and Xinyue capsule (100 mg/day). 24 weeks. Monitoring ADRs during trials.	Xinyue capsule added on conventional treatment reduced the incidence of cardiac death, nonfatal myocardial infarction, and urgent revascularization.	[199]

Abbreviation: Monitor New York Heart Association (NYHA); 6 min walking distance, short-form 36 (SF-36); off-pump coronary artery bypass graft (OPCABG). blood pressure (SBP), heart rate (HR), blood pressure (MBP), and diastolic blood pressure (DBP). ↓: Decrease; ↑: Increase.

## 6. Conclusions

ANTs are important in the treatment of solid tumors and hematological neoplasms, although their practical clinical application is greatly limited because of their AIC production. Oxidative stress, mitochondrial dysfunction, and various forms of cell death, such as apoptosis, ferroptosis, and pyroptosis, can mediate the development of AIC [5]. Currently, there is an urgent need for clinical drugs that can protect the heart, are safe and stable, and do not interfere with the antitumor effects of ANTs. Ginsenosides, which can strengthen the heart, improve cardiac function, and inhibit vascular damage and MI, are considered highly important for cardiac protection [200]. This review concludes that ginsenosides can alleviate myocardial injury induced by oxidative stress through the regulation of multiple pathways, including the Nrf2/ARE, MAPK, and STAT3 phosphorylation pathways and the PI3K/Akt, NF-κB, and SIRT1 pathways. In addition, through the mechanisms underlying the development of AIC, ginsenosides have been shown to ameliorate myocardial injury through multiple pathways. These include increasing the mitochondrial DNA content to restore mitochondrial function; regulating calcium ion metabolism; inhibiting cardiomyocyte pyroptosis, apoptosis, and ferroptosis; and modulating autophagy. However, few studies have investigated the role of ginsenosides in AIC, particularly through the inhibition of pyroptosis and the regulation of autophagy. Therefore, this article reviews research on the cardioprotective effects of ginsenosides in other CVDs via these mechanisms, aiming to broaden the therapeutic scope and provide a foundational basis for future clinical applications in AIC. Taken together, these findings indicate that ginsenosides utilize a multitarget and multipathway mechanism for the prevention and treatment of AIC. Although ginsenosides have multidimensional protective potential for the prevention and treatment of AIC, there is currently a lack of evidence supporting their clinical application. Moreover, with the continuous exploration of novel ginsenosides, the common mechanisms through which different ginsenosides treat AIC need to be further investigated. Future research could focus on integrating multiomics techniques to elucidate the precise targets of action involved in this process. Moreover, enhancing the safety and robustness of ginsenosides through studies would facilitate the effective translation of basic research to clinical application. This will provide a new direction, ensuring both efficacy and low risk, for the prevention and treatment of AIC and other CVDs.

## Figures and Tables

**Table 1:** Classification of ginsenosides.

Class		-OR of Points	Representative Ginsenosides
Dammarane type	Protopanaxadiol (PPD)	C3; C20	C-K, Rd, Rg3, Rb, Ra1, Ra2, Ra3, Rb, Rc, Rd, Rg3, Rh1, Rh2, Rh3, Rh4, F2
	Protopanaxatriol (PPT)	C6; C20	Re, Rf, F1, F3, F4, F5, Rg1, Rg2, Rh1, notoginsenoside R1
Ocotillol type		C24; C20	ginsenoside R2, notoginsenoside R1, pseudoginsenoside F11 Makonoside-Rs
Oleanolic acid type			Ro, Ri

**Table 4:** Ginsenosides treat CVDs by regulating autophagy.

Animal Model	Treatment Protocol	Autophagy Marker Change	Effects of Autophagy Targeting	Reference
Male C57BL/6 mice. 8–10 weeks old. DOX 20 mg/kg i.p. weekly.	Gavage administration of Rb1 (40 mg/kg/day).	LC3-I, p62 ↓.	Inhibiting autophagy	[116,122]
H/R treatment, H9c2 cardiomyocytes	Rg1 (100 μmol/L), 24 h.	LC3-II, Beclin-1, p62 ↓, inhibiting AMPK pathway.	Inhibiting autophagy	[123]
Male rats weighing 280–320 g. Balloon-injury.	Gavage administration of Re 12.5/25 mg/kg, 2 weeks.	ERK1/2, LC3-I, p62 ↓	Inhibiting autophagy	[124]
Mouse primary peritoneal macrophages. ox-LDL (100 μg/mL)–24 h.	Ox-LDL and 10/20/40/80 μM Rb1(24 h).	AMPK, LC3-II ↑; SQSTM1/p62 degradation.	Rb1 rescues autophagy flux, inducing autophagy.	[125]
ICR male mice. Coronary artery ligation (CAL).	Rb1 6 mg/kg i.p. after 20 min of CAL.	PINK1,Parkin,LC3-II/LC3-I ↑, p62 ↓ to increase degradation.	Rb1 exerts cardioprotective functions through activation of mitophagy via AMPKα pathway.	[126]
C57BL/6 male mice (9–11 weeks old). Left anterior descending coronary artery (LAD). 28 days.	Gavage administration of Rg1 (20 mg/kg).	LC3-II ↑, p62 ↓ GRg1 significantly increases SIRT1 expression and activates the PINK1/Parkin signaling pathway.	Rg1 enhances mitochondrial autophagy and alleviates HF.	[127]
Male Sprague Dawley (SD) rats. The left anterior descending branch-ligated HF rat model. OGD/R H9c2 cell model.	Gavage administration of Rb2 (10 mg/kg, 20 mg/kg, daily for 3 days)	miR-216a-5p ↓, LC3B II/I, Beclin1 ↑.	Rb2 promotes autophagy treatment for HF.	[130]

Abbreviation: Oxygen Glucose Deprivation/Reperfusion (OGD/R). ↓: Decrease; ↑: Increase.

## Data Availability

No new data were created or analyzed in this study.

## Abbreviations

TfR	Transferrin Receptor	Akt	protein kinase B
FTH1	Ferritin Heavy Chain 1	LC3II	light chain 3II
IRP1	Iron Regulatory Protein 1	Atg	autophagy-related gene
Bcl-2	B cell leukemia-2	caspase	Cysteine-aspartic protease
NOS	Nitric Oxide Synthase	Mdm2	Mouse double minute 2 homolog
GSH	glutathione	CK	creatine kinase
SOD	superoxide dismutase	LDH	lactate dehydrogenase
ATP	adenosine triphosphate	GPx	glutathione peroxidase
eNOS	endothelial nitric oxide synthase	HO-1	heme oxygenase 1
PI3K	phosphatidylinositol 3-kinase	AST	aspartate transaminase
TGF-β	transforming growth factor-β	COL-IV	Collagen Type IV
VEGF	Vascular Endothelial Growth Factor	MPO	Myeloperoxidase
TNF-α	tumor necrosis factor-α	G-CSF	Granulocyte Colony-Stimulating Factor
MMP	Matrix Metalloproteinases	GDH	Glutamate Dehydrogenase
α-SMA	α-Smooth Muscle Actin	TLR4	Toll-like receptor 4
FAK	Focal Adhesion Kinase	SIRT1	sirtuin 1
p62	ubiquitin-binding protein p62	A20	A20 Zinc Finger Protein
GPX4	Glutathione Peroxidase 4	SIRT3	sirtuin 3
ANP	atrial natriuretic peptide	MyHc	myosin heavy chain
β-MHC	β-myosin heavy chain	4-HNE	4-Hydroxy-2-nonenal
GSSG	Glutathione disulfide	Cyt-c	cytochrome c
NF-κB	Nuclear Factor-Kappa B	Col	Collagen type
PARP	Poly (ADP—Ribose) Polymerase	LC3	light chain 3
JNK	c-Jun N-terminal Kinase	MFN2	Mitofusin 2
AP-1	Activator Protein-1	Collagen I	Collagen Type I
PINK1	PTEN induced putative kinase 1	NOX	NADPH oxidase
GSK-3β	Glycogen synthase kinase 3β	ER	Estrogen Receptor
ARE	Antioxidant Response Element	ERK	Extracellular Signal-Regulated Kinases
RhoA	Ras homolog gene family, member A	MIP-1δ	Macrophage Inflammatory Protein-1δ
Bax	Bcl-2-associated X apoptosis regulator	H-keap1	kelch-like ECH-associated protein 1
ICAM-1	Intercellular Adhesion Molecule-1	Bad	Bcl-2 associated death promoter
VPS	Vacuolar protein sorting 34	HIF-1α	Hypoxia-Inducible Factor-1α
MAPK	Mitogen-Activated Protein Kinase	Smad 2/3	Mothers against decapentaplegic homolog 2/3
Ras	Rat sarcoma virus oncogene homolog	EHA	European Hematology Association Guidelines
NIX	Nip3—like protein X	FUNDC1	FUN14 Domain—containing 1
SLC7A11	Solute Carrier Family 7 Member 11	FIP200	200—kDa FAK—family interacting protein
COL1A1	Collagen Type I Alpha 1 Chain	ATG14L	Autophagy—related 14
FADD	Fas—associated death domain protein	SLC7A11	Solute Carrier Family 7 Member 11
mTOR	mechanistic target of rapamycin	ETC	Electron Transport Chain
DMT1	Divalent Metal Transporter 1	FPN	Ferroportin
PUMA	p53—upregulated modulator of apoptosis	BNIP3	Bcl—2/adenovirus E1B 19kDa—interacting protein 3
ESC	European Society of Cardiology Guidelines	PGC-1α	Peroxisome proliferator-activated receptor gamma coactivator 1α
TRADD	Tumor Necrosis Factor Receptor 1—associated Death Domain protein	ABCB1	ATP—Binding Cassette Sub—family B Member 1
Nrf2	nuclear factor erythroid 2-related factor 2	AMPK	adenosine monophosphate-activated protein kinase
NLRP3	NOD-like receptor protein-3 GSDME:Gasdermin E	ASC	Apoptosis-associated speck-like protein containing a CARD
ROCK	Rho-associated coiled-coil containing protein kinase	NOXA	Novel Oxidation—inducible protein in Astrocytes
NADPH	Nicotinamide Adenine Dinucleotide Phosphate Hydrogen	CSCO	Chinese Society of Clinical Oncology Guidelines
SERCA	Sarcoplasmic/Endoplasmic Reticulum Calcium ATPase	LOX-1	Lectin-like Oxidized Low-Density Lipoprotein Receptor-1
Apaf1	Apoptotic Peptidase Activating Factor 1	ULK1	unc—51 like autophagy activating kinase 1
